# Novel Inhibitor of Keap1-Nrf2 Protein–Protein Interaction Attenuates Osteoclastogenesis In Vitro and Prevents OVX-Induced Bone Loss In Vivo

**DOI:** 10.3390/antiox13070850

**Published:** 2024-07-15

**Authors:** Zhihao Chen, Hongyuan Yao, Alessandra Marie Encarnacion, Jujin Jeong, Yunju Choi, Sangwook Park, Sunwoo Lee, Taehoon Lee

**Affiliations:** 1 Department of Oral Biochemistry, Dental Science Research Institute, School of Dentistry, Chonnam National University, Gwangju 61186, Republic of Korea; chinaczhihao@jnu.ac.kr (Z.C.); swpark@chonnam.ac.kr (S.P.); 2 Department of Interdisciplinary Program of Biomedical Engineering, School of Dentistry, Chonnam National University, Gwangju 61186, Republic of Korea; chinayao@jnu.ac.kr (H.Y.); amencarnacion@jnu.ac.kr (A.M.E.); ju0203@jnu.ac.kr (J.J.); 3 Department of Dental Bioscience, School of Dentistry, Chonnam National University, Gwangju 61186, Republic of Korea; 207764@jnu.ac.kr; 4 Department of Chemistry, Chonnam National University, Gwangju 61186, Republic of Korea; sunwoo@jnu.ac.kr

**Keywords:** Keap1, osteoclastogenesis, Nrf2, antioxidants, ROS

## Abstract

Keap1 interacts with Nrf2 by assisting in its ubiquitination and subsequent proteolysis. By preventing ROS accumulation during RANKL-induced osteoclastogenesis, Nrf2 activation can prevent the differentiation of osteoclasts. Additionally, inhibiting the Keap1-Nrf2 PPI can be an effective strategy for triggering Nrf2 to regulate oxidative stress. Structure-based virtual screening was performed to discover a potentially novel Keap1-Nrf2 PPI inhibitor wherein KCB-F06 was identified. The inhibitory effects of KCB-F06 on osteoclastogenesis were investigated in vitro through TRAP staining and bone resorption assays. An ovariectomy-induced osteoporosis mouse model was applied to evaluate KCB-F06’s therapeutic effects in vivo. Lastly, the underlying mechanisms were explored using real-time PCR, Western blotting, and co-IP assays. KCB-F06 was discovered as a novel Keap1-Nrf2 PPI inhibitor. As a result, the expression of antioxidants (HO-1 and NQO1) was suppressed, hence reducing ROS accumulation during osteoclastogenesis. Subsequently, this caused the inactivation of RANKL-induced IKB/NF-kB signaling. This eventually led to the downregulation of osteoclast-specific proteins including NFATc1, which is an essential transcription factor for osteoclastogenesis. These results demonstrated that Nrf2 activation in osteoclasts is a valuable tool for osteoclastic bone loss management. In addition, KCB-F06 presents as an alternative candidate for treating osteoclast-related bone diseases and as a novel small molecule that can serve as a model for further Keap1-NRF2 PPI inhibitor development.

## 1. Introduction

Osteoporosis is a systemic disease associated with dysregulated bone resorption and osteogenesis. Serious osteoporosis increases the risk of fracture, especially in adults aged over 50 years [1]. With a global aging population, osteoporosis has become a major health concern around the world [2,3]. Osteoporosis is known to develop in postmenopausal women due to un-balanced bone homeostasis characterized by osteoclast-mediated bone resorption activity being higher than the osteoblast-mediated bone generation [2,4]. In the current clinical setting, bisphosphonates are the first-line drugs being used for treating osteoclastic bone loss, while denosumab and raloxifene, antibody and hormone therapy drugs, respectively, have been licensed for treating osteoporosis and osteoporotic bone fractures [1,5]. However, these currently in-use drugs present several potential drawbacks. Bisphosphonate treatment increases fracture or crack risks in the mid-thighbone as well as induced jaw necrosis. Moreover, raloxifene can cause blood clots and increase the risk of cancer after long-term treatment [1,6]. Hence, the discovery of more effective, new, and safer anti-osteoporosis chemicals has become a pressing issue.

Osteoclasts play a crucial role in the occurrence and progression of osteoporosis. In a healthy bone remodeling situation, there is a delicate balance between osteoblastic matrix synthesis and osteoclastic matrix resorption [2,4,7]. Conversely, osteoporosis represents a systemic skeletal pathological imbalance characterized by diminished bone mineral density and perturbations in the bone microstructure, resulting in a heightened susceptibility to fractures [2,4,7,8].

Reactive oxygen species (ROS), a by-product during RANKL-induced osteoclastogenesis, play a vital role during osteoclast differentiation and its function [9]. ROS have the ability to trigger the activation of downstream signaling pathways such as I-κb/NF-κb, which are not just engaged in osteoclast formation but also in osteoclast function [10]. Several antioxidants (terpenoids, praelolide, and curcumin) have proven that reducing ROS can prevent the formation of osteoclasts and bone resorption [9]. Hence, the management of imbalanced ROS generation and accumulation may be a useful strategy for treating osteoclast-related diseases [11].

Nuclear factor-erythroid 2-related factor 2 (Nrf2) transcribes the expression of many antioxidants and phase II detoxifying enzymes. It is also referred to as a redox-sensitive basic leucine zipper transcription factor that binds the antioxidant response element, a cis-acting enhancer sequence, to regulate the generation and buildup of ROS [12]. The overexpression of Nrf2 or Nrf2 activators inhibited osteoclastogenesis, whereas the pharmacological inhibition of Nrf2 or knockout of Nrf2 induces this process [10,13]. Inhibition of Keap1-Nrf2 PPI was discovered as a new approach to regulate oxidative stress and prevent or treat oxidation-stimulated diseases by activating Nrf2 [14].

Therefore, to contribute to the discovery of novel anti-osteoporosis agents, we identified KCB-F06 as a novel Keap1–Nrf2 PPI inhibitor that showed anti-osteoclastogenic activity in vitro and blocked OVX-mouse bone loss in vivo. Mechanistic studies also demonstrated that KCB-F06 directly interrupted Keap1-Nrf2 PPI by targeting Keap1, hence activating the Nrf2 protective pathway for anti-osteoclastogenesis. We concluded that KCB-F06 could be a novel agent that inhibits osteoclast differentiation for treating osteoclastic bone loss by the activation of Nrf2.

## 2. Materials and Methods

### 2.1. Reagents

KCB-F06, PubChem CID: 2915130; N-[2-methyl-4-(phenylsulfanylmethyl)phenyl]-2-(N-methylsulfonylanilino)propanamide, was obtained from Chembridge and dissolved in dimethyl sulfoxide (DMSO; Sigma-Aldrich, St. Louis, MO, USA). Tartrate-resistant acid phosphatase staining kit was obtained from CosmoBio. The fetal bovine serum (FBS) and α-modification of the Eagle minimal essential medium (α-MEM) were purchased from Gibco (Grand Island, NY, USA). Murine M-CSF and RANKL were purchased from PeproTech (Cranbury, NJ, USA). Anti-β-actin (A5441) was bought from Sigma-Aldrich. Anti-cathepsin K (sc-48353) was obtained from Santa Cruz Biotechnology (Dallas, TX, USA). Anti-c-fos (4384s), anti-NFATc1 (8032s), and secondary antibodies (7074s and 7076s) were supplied by CST. The ECL system (RPN2235) for detecting chemiluminescence signals was purchased from iNtRON (Essex, UK).

### 2.2. Isolation and Culture of Bone Marrow-Derived Macrophages

After flushing the femurs and tibias of 8-week-old C57BL/6J female mice with α-MEM, bone marrow cells (BMCs) were collected. Following that, the BMCs were cultured for 24 to 36 h at 37 °C with 5% CO_2_, α-MEM in 10% FBS, 100 U/mL of penicillin, and 100 μg/mL of streptomycin in an incubator. After being separated from the supernatant, the cells were grown for three days with a continuous supply of 30 ng/mL M-CSF. The adhering cells were then extracted and used to create bone marrow-derived macrophages (BMDMs), which were then used to induce osteoclasts [15].

### 2.3. Culture of Human Osteoclast from hiPSC-Derived Monocyte

hiPSCs were maintained in mTeSR1 medium on matrigel-coated 6-well cell culture plate and passaged by using Accutase (San Diego, CA, USA). Cells were incubated at 37 °C, 5% CO_2_.

iPSCs were washed with PBS, incubated in Accutase for 5 min at 37 °C, 5% CO_2_, and then lifted in DMEM/F12 to obtain single cell suspension. Cells were pelleted by centrifugation at 1200 rpm for 3 min. Subsequently, cells were resuspended in 1 mL mTeSR1 medium and counted. Moreover, 2.5 × 10^4^ cells were seeded per well in a round bottom ultra-low attachment 96-well cell culture plate in 100 µL mTeSR1 medium supplemented with 50 ng/mL human bone morphogenetic protein 4 (hBMP4), 50 ng/mL human vascular endothelial growth factor (hVEGF), 20 ng/mL human stem cell factor (hSCF), and 10 µM Rock-inhibitor Y-27632. The plate was centrifuged at 1500 rpm for 10 min. Half change to culture medium on day1 and day 2. 4 days after, EBs were transferred to 6-well cell culture plates coated 0.1% gelatin (16 EBs/well) and containing 3 mL/well differentiation medium (X-VIVO15 medium, GlutaMAX Supplement, 2-Mercaptoethanol, 1% Penicillin/Streptomycin, 25 ng/mL human interleukin 3[hIL-3], 100 ng/mL human macrophage colony-stimulating factor[hM-CSF]). Produced monocyte-like suspension cells were harvested every 2 days and replaced with 2 mL fresh medium [15,16,17].

Monocyte-like cells were filtered and harvested to obtain a single cell. Cells were pelleted by centrifugation at 1200 rpm for 3 min. Subsequently, cells were resuspended in 1 mL X-VIVO15 medium and counted. Moreover, 2 × 10^4^ cells were seeded per well in 96-well cell culture plate in 200 µL/well induction medium X-VIVO15 medium, GlutaMAX Supplement, 2-Mercaptoethanol, 1% Penicillin/Streptomycin, 100 ng/mL human granulocyte-macrophage colony-stimulating factor[hGM-CSF]). 3 days after, change to osteoclasts differentiation medium (αMEM, 10% FBS, 1% Pen/Strep, 50 ng/mL hM-CSF, 100 ng/mL human soluble receptor activator of nuclear factor-κ B ligand[hsRANKL]). The cells were cultured for an additional 7 to 14 days. Every 2 days, half of the medium was replaced with fresh osteoclast differentiation medium [15,16,17].

### 2.4. In Vitro Osteoclast Culture, TRAP Staining, and Cell-Viability Assay

BMDMs, the attached cells, were harvested and cultured in α-MEM containing 10% FBS, 100 U/mL of penicillin, 100 μg/mL of streptomycin, 30 ng/mL of M-CSF, and 50 ng/mL of RANKL for 3-5 days, with or without KCB-F06 incubation. Medium change was performed every 2 days. Once the positive control group formed mature osteoclasts, they were fixed using 4% paraformaldehyde (PFA) for approximately 20 min at room temperature (RT). Then, the osteoclasts were stained by using a TRAP-staining kit. The spread osteoclast areas were visualized using a microscope and calculated using ImageJ (https://imagej.net/software/fiji/downloads, accessed on 1 December 2023); the number and spread area of TRAP-positive cells with 3 or more nuclei were considered mature osteoclasts. Using a cell viability test kit, the BMDMs (2 × 10^4^ cells/well) were evaluated for viability in a 96-well plate, as previously described.

### 2.5. Osteogenesis and Alkaline Phosphatase (ALP) Staining Assay

Three to four-day-old mice’s calvarias were used to extract primary osteoblasts. The cells were incubated for nine days in α-MEM with 10% FBS and 1% penicillin-streptomycin solution, either with or without KCB-F06 and BMP2 (100 ng/mL). Every two days, the medium was changed. After three times of PBS washing, the cells were fixed for one hour in 70% ice-cold ethanol, and then they were twice rinsed with double-distilled H_2_O. Next, the cells were stained using a BCIP^®^/NBT liquid substrate system for 5–15 min at RT, as performed previously. The images were captured with a microscope or through a direct scan. The intensity of ALP-positive cells was determined using ImageJ (https://imagej.net/software/fiji/downloads, accessed on 1 December 2023). [18].

### 2.6. Bone Resorption and F-Actin Belt Staining Assay

The bone resorption assay was performed as previously described. First, 2 × 10^4^ BMDMs per well were cultured in the supplied kit (CSR-BRA-48X2KIT) with a fluorescein amine-labeled calcium phosphate-coated 48-well plate. After that, KCB-F06 was incubated with or without the indicated dose until mature osteoclasts were visible. According to the instructions provided by the kit we are using, the coated calcium phosphate is first bound to fluoresceinamine-labeled chondroitin sulfate (FACS), which is released from the calcium phosphate layer into the conditioned medium by osteoclastic resorption activity. Bone resorption activity is evaluated by simply measuring the fluorescence intensity of the conditioned medium. Next, to assess the fluorescence intensity, 100 μL of the supernatant from each well was collected into a 96-well black polypropylene microplate. The SpectraMax i3x fluorescence plate reader was used at an excitation wavelength of 485 nm and an emission wavelength of 535 nm. A microscope was used to view the resorbed pit area, which was then calculated using ImageJ (https://imagej.net/software/fiji/downloads, accessed on 1 December 2023). Regarding the F-actin ring assay, the belts were visible with a rhodamine-conjugated phalloidin staining kit (Abcam, Cambridge, UK), as previously described [18].

For the f-actin belt staining assay, BMMs after treating KCB-F06 for 3–5 days (the control group formed mature osteoclasts), the cells were fixed in 4% PFA for 15 min (RT) and permeabilized with 0.5% Triton X-100 for 40 min (RT). After washing with 1X PBS, F-actin belts were stained using rhodamine phalloidin (cytoskeleton) diluted in 2.5% bovine serum overnight at 4 °C. Then, DAPI staining (5 min) was performed to visualize the nuclei. Finally, the F-actin rings were visualized under the fluorescence microscope (Olympus IX73) [18].

### 2.7. Detection of ROS Production

Using a DCFDA/H2DCFDA-Cellular ROS Assay Kit (Abcam), the levels of intracellular ROS were measured. Following a 3-day treatment period for RANKL with or without varying dosages of KCB-F06, the cells were cultured using the fluorescent probe (20 μM H2DCFDA) in a serum-free medium in accordance with the manufacturer’s guidelines. Leica TCS SP5 AOBS laser scanning confocal microscope (Zeiss, Oberkochen, Germany) was used to take pictures of ROS-positive cells [9,13,19].

### 2.8. RNA Isolation and Quantitative Real-Time PCR

Total RNA was extracted from cells using Trizol, as described previously. After quantifying the concentrations of RNA, they were reverse transcribed to cDNA using a PrimeScript RT Reagent Kit (Takara, San Jose, CA, USA) following the manufacturer’s instructions. Next, the cDNA was used for real-time PCR amplification reactions by using Power SYBR Green PCR Master Mix. The mouse glycer-aldehyde-3-phosphate dehydrogenase (GAPDH) gene was used as a reference gene. The primer sequences for the qRT-PCR are listed in Table S1 [18].

### 2.9. Cellular Thermal Shift Assay (CETSA)

To confirm the interaction between KCB-F06 and Keap1, CETSA experiments were performed. BMDMs were harvested and lysed with CelLyticTM M (C2978-250ML) supplemented with protease and phosphatase inhibitor. The lysate was divided into two groups and treated with DMSO or KCB-F06 for 1 h at RT. Then, the lysates from the two groups were separated into smaller aliquots and heated separately at various temperatures (37, 45, 50, 55, 60, 65, 70, 75 °C) for 5 min, followed by cooling on ice. After centrifuging the heated lysates for 20 min at 4 °C at 13,000 rpm, further Western blot analysis was performed with this group of samples [20,21].

### 2.10. Drug Affinity Responsive Target Stability (DARTS)

BMDMs were cultured in 10 cm dish for three days, then harvested and lysed with CelLyticTM M supplemented with protease and phosphatases inhibitor. In brief, protein samples from BMDMs were divided into four groups: each group of 297 μL protein was added with 3 μL DMSO or different concentrations of KCB-F06 and incubated at RT for 1 h. The four groups of samples were then digested with pronase solution (roche, Basel, Switzerland) [pronase:protein = 1:200 (*w*/*w*)] for 30 min, and the digestion reaction was stopped with 4 X SDS loading buffer at the same time and then immediately boiled at 95 °C for 10 min. A Western blot assay was performed to detect the protection of KCB-F06 on Keap1 degradation [15,22].

### 2.11. Western Blotting and Immunoprecipitation Assay

Cells were lysed with RIPA buffer mixing protease inhibitor. Nuclear proteins were extracted using an extraction kit (#78835, Thermo Fisher Scientific, Waltham, MA, USA). Protein concentrations were determined using a BCA kit (A53225 Thermo Fisher Scientific). After boiling at 95 °C for 10 min, the same amounts of proteins were subjected to Western blot analysis using 8% or 12% SDS-PAGE as previously. Primary antibodies used were Anti-Nrf2 (#16396-1-AP, Proteintech, Rosemont, IL, USA), Anti-Ubiquitin (#3936s, CST), anti-NQO1 (#ab34173), Anti-Keap1 (#10503-2-AP, Proteintech), anti-HO-1 (#ab13243) from Abcam, Cambridge, UK. anti-phospho-NF-κB (#S536), anti-NF-κB (#4764s), anti-GAPDH (#5174S) anti-phospho-IκBa (#2859s), and anti-IκBa (#9242s) from CST, Boston, MA, USA. Anti-rabbit (#7074S, CST) and anti-mouse (#7076S, CST) were used as HRP-conjugated secondary antibodies. Finally, a super plus enhanced chemiluminescence (ECL) buffer was used to visualize target bands for imaging. Using ImageJ (https://imagej.net/software/fiji/downloads, accessed on 1 December 2023), the gray levels of the bands were measured [23,24]. Co-immunoprecipitation was carried out as previously mentioned. In brief, BMDMs were cultured in 6-well plates for 2 days with M-CSF, RANKL, and KCB-F06. Cells were lysed in IP lysis buffer and then centrifuged at 13,000 rpm for 20 min at 4 °C; the supernatant was considered a protein sample. After quantifying different sample protein concentrations with BCA, Super-protein A/G beads (sc-2003, Santa Cruz, CA, USA) were then incubated with the resulting protein samples (300 µL) overnight at 4 °C. Samples were premixed with anti-Keap1 antibody (2 µL) 1 h before the beads were mixed. The corresponding species of IgG was added, and the same process was performed with the primary antibody as a control group. Beads were collected by gentle centrifugation and the supernatant was carefully discarded. The beads were washed 5 times with IP lysis buffer. Then, the pellets were boiled at 95 °C in 1X SDS buffer. The protein was analyzed by Western blotting as described above [9,15,19,25].

### 2.12. Molecular Docking

Using AutoDock Vina, molecular docking was used for virtual Keap1-Nrf2 PPI inhibitor screening. In brief, the Protein Data Bank provided the crystal structure of Keap1. (PDB) (ID: 7K2C, human). A total of 1671 structures available on Korea Chemical Bank were prepared for virtual screening [26,27]. The Keap1-Nrf2 PPI binding interface that was utilized for screening was found to have been previously reported [26,27]. Using semi-flexible docking with AutoDock Vina, the binding affinity of drugs with human Keap1 was determined in the main virtual screening [18]. After the screening, the selection between candidate inhibitors with binding affinity below −7 kcal/mol was made based on the synthetic difficulty, purchasing availability, and price.

### 2.13. OVX-Induced Osteoclastic Bone-Loss Mice Model

The C57BL/6J mice were raised in a dedicated environment free of pathogens. As previously mentioned, we established a model of osteoporosis in mice induced by ovariectomy (OVX) to investigate the preventive effects of KCB-F06 in vivo [15]. First, 7-week-old C57BL/6J female mice were grouped into three groups: OVX, Sham, and KCB-F06 (20 mg/kg). While the mice in the Sham group simply had an abdominal incision, the animals in the OVX and KCB-F06 groups underwent OVX surgery. After one-week recovery, the KCB-F06 group received an intraperitoneal injection with 20 mg/kg of a mixture comprising 10% Tween 80 and 10% DMSO, while the mice of the Sham and OVX groups injected an equal volume of 10% Tween 80 and 10% DMSO intraperitoneal injections daily for four weeks. The total injection volume was 200 µL per mouse. The mice were weighed weekly. In preparation for microcomputed tomography (micro-CT) analysis, the mice were euthanized on the last day of the experiment, and their femurs were separated and preserved in 4.0% paraformaldehyde. For histological investigation, all the fixed tibias and femurs were subsequently decalcified for four weeks using 10% ethylenediaminetetraacetic acid (EDTA). Following the paraffin sectioning process, the various sample slices were stained using a TRAP kit, hematoxylin, and eosin (H&E).

### 2.14. Micro-CT Scanning

The Quantum GX Micro-CT imaging system at Korea Basic Science Institute (Gwangju, Republic of Korea) was used for micro-CT scanning, as in our previous studies [18,23].

### 2.15. Subsection Statistical Analysis

The differences between the two groups were compared using Student’s *t*-test. GraphPad Prism 9.4.0 was utilized to perform a one-way analysis of variance (ANOVA) with Dunnett’s multiple comparison tests to analyze differences between several groups. (* *p* < 0.05; ** *p* < 0.01; *** *p* < 0.001; NS, not significant). All error analysis was expressed as means ± SD.

## 3. Results

### 3.1. Structure-Based Virtual Screening for the Discovery of KCB-F06 as a Novel Keap1-Nrf2 PPI Inhibitor

**Figure 1:**
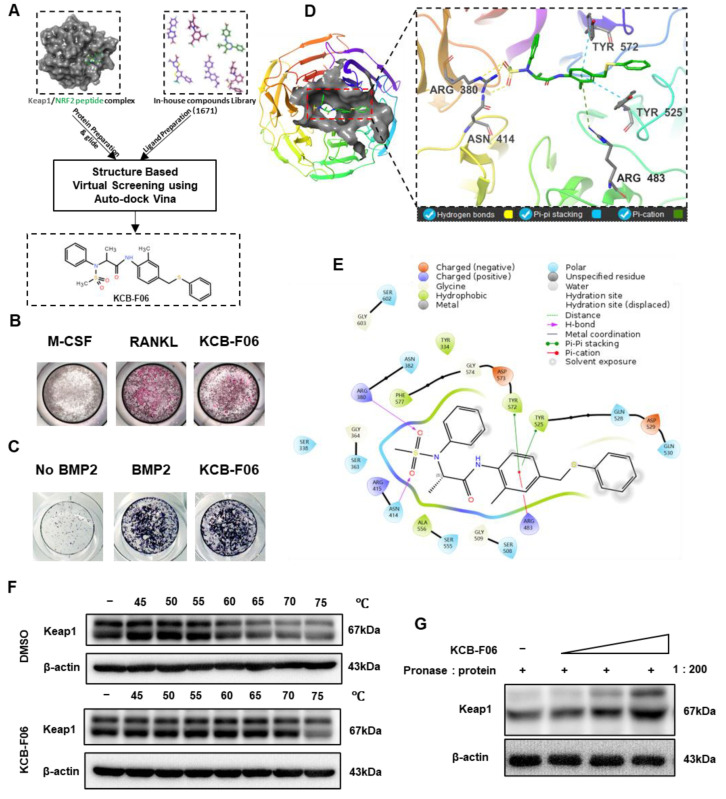
Screening and identification of KCB-F06 as an anti-osteoclast inhibitor by targeting Keap1. (**A**) A structure-based virtual screening assay using AutoDock Vina (1.1.2) led to the identification of KCB-F06 as a candidate for inhibiting the Keap1-Nrf2 PPI from 1671 in-house compound library. (**B**) TRAP and (**C**) ALP staining was employed to confirm the impact of KCB-F06 on osteoclastogenesis and osteogenesis, respectively. (**D**,**E**) The anticipated binding pose and interactions between KCB-F06 and Keap1 (PDB ID: 7K2C) are illustrated, along with a corresponding 2D interaction diagram generated from Maestro. (**F**) CETSA and (**G**) To confirm the direct interaction between Keap1 and KCB-F06 in vitro, DARTS assays were performed. *n* = 3.

To discover a novel Keap1-Nrf2 PPI inhibitor, a structure-based virtual screening assay using AutoDock Vina was performed. This led to the discovery of KCB-F06 as a potential candidate due to its advantages over other putative inhibitors with binding affinity below −7 kcal/mol, namely, ease of synthesis, purchasing availability, and price (Figure 1A). Next, the TRAP staining assay (a method that identifies osteoclast differentiation) and ALP staining assay (a method that identifies osteogenesis) were performed to confirm the effects of KCB-F06 on osteoclastogenesis and osteoblast differentiation, respectively. As shown in Figure 1B,C, the 2 µM treatment of KCB-F06 inhibited osteoclastogenesis, while it did not influence osteogenesis. Meanwhile, Figure 1D displayed the best binding pose between Keap1 and KCB-F06. Results suggested that KCB-F06 binds with Keap1 by forming triple hydrogen bonds with ARG380 and ASN414 residues. Two Pi-Pi stackings were also formed between KCB-F06 and Keap1 (TYR 572 and TYR 525). In addition, a Pi-cation interaction has also been suggested to be formed between KCB-F06 and the ARG483 residue of Keap1 (Figure 1D,E). Lastly, the direct interaction of KCB-F06 and Keap1 was further confirmed in vitro using DARTS and CETSA assays (Figure 1F,G).

### 3.2. KCB-F06 Suppressed RANKL-Induced Osteoclastogenesis In Vitro

**Figure 2:**
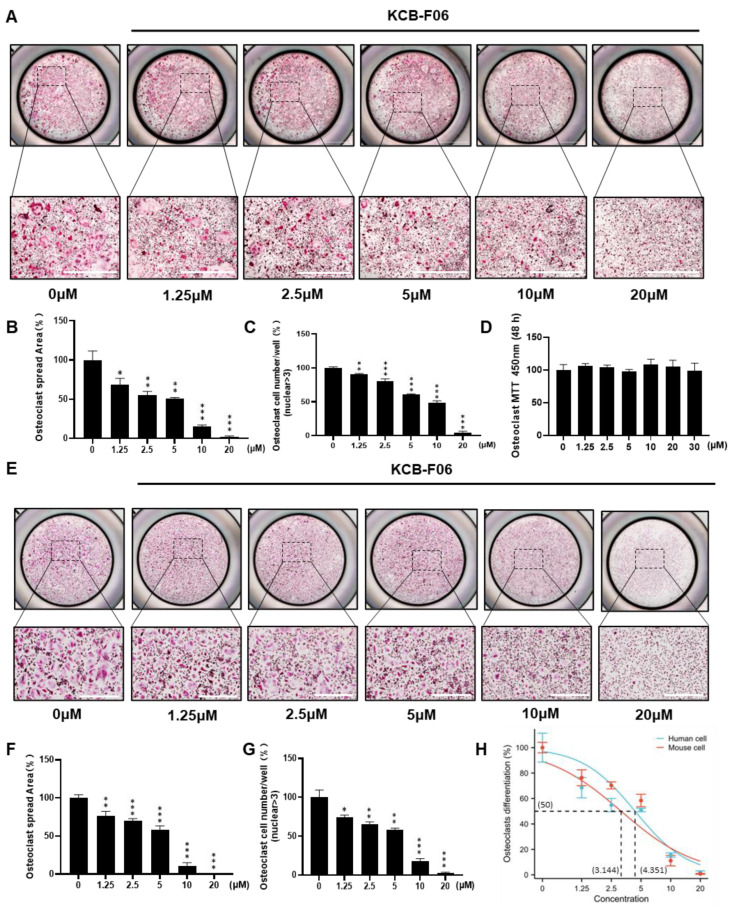
KCB-F06 inhibits both mouse and human osteoclastogenesis in vitro. (**A**) Representative TRAP staining images of mouse OCs after treatment with varying concentrations of KCB-F06. Scale bar = 1000 μm. *n* = 4. (**B**) The quantitative analysis of TRAP-positive multinucleated cells area (nuclei > 3) per well and (**C**) osteoclast cell number. (**D**) Effect of KCB-F06 on BMDM proliferation. The indicated doses were incubated with BMDMs for 48 h. Cell viability was determined by MTT assay. (**E**) Representative TRAP staining image of iPSC-induced human OCs after treatment with different concentrations of KCB-F06. *n* = 4. (**F**) The quantitative analysis of TRAP-positive multinucleated cells area (nuclei > 3) per well and (**G**) osteoclast cell number. (**H**) The TRAP-positive area of the panel (**B**) was used for calculating the IC50 of KCB-F06 on osteoclastogenesis. *** *p* < 0.001 vs. control, ** *p* < 0.01 vs. control, * *p* < 0.05 vs. control.

Next, the inhibitory effect of KCB-F06 on osteoclastogenesis was further investigated. As shown in Figure 2A,E, KCB-F06 inhibited the differentiation of both mouse BMDMs and human IPSC-derived osteoclasts. The osteoclast spread area and osteoclast cell number per well were calculated and displayed in Figure 2B,C (mouse OCs) and Figure 2F,G (human OCs). Results suggested that KCB-F06 inhibited both mouse and human osteoclast differentiation in vitro in a dose-dependent manner. To determine whether KCB-F06 has cytotoxicity in its working concentrations after KCB-F06 was treated for 48 h at the indicated concentrations, the MTT assay was performed in BMDMs (Figure 2D). The optical density readouts demonstrated that KCB-F06 shows no toxicity at concentrations lower than 30 µM. Finally, 3.144 ± 0.379 and 4.351 ± 0.42 µM were the calculated IC50 values of KCB-F06 on mouse and human osteoclast differentiation, respectively (Figure 2H). BMDMs were administered with KCB-F06 (5 and 10 μM) during the relevant period of osteoclast differentiation to see if KCB-F06 would affect osteoclast differentiation in a time-dependent manner (Figure S1A–C). Results suggested that the suppression of osteoclastogenesis by KCB-F06 occurred mainly at the early to middle stages of osteoclast differentiation (Figure S1A–C). Furthermore, we investigated the potential impact of KCB-F06 on osteoblasts, revealing that it does not exert a significant effect on osteoblasts at concentrations up to 40 μM (Figure S2).

### 3.3. KCB-F06 Inhibited F-Actin Belt Formation and Bone Resorption

**Figure 3:**
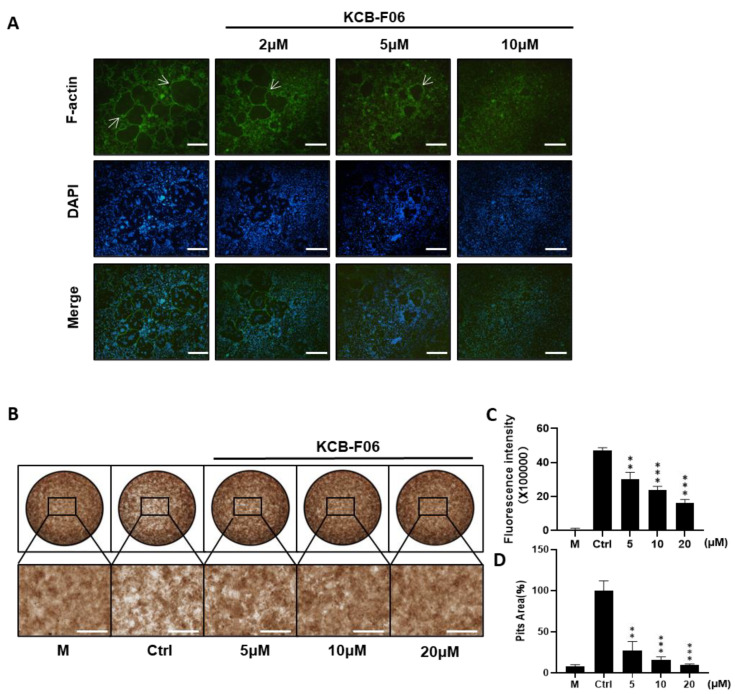
KCB-F06 inhibits the bone resorption activity of osteoclasts in vitro. (**A**) Representative images of F-actin rings after BMMs were treated with varying concentrations of KCB-F06 or Alendronate. *n* = 4. The white arrow points to the edge of F-actin positive osteoclasts. (**B**) Images showing the concentrations of KCB-F06 and representative bone resorption pits in each group treated with or without RANKL after BMMs were cultured in a 48-well fluorescein amine-labeled calcium phosphate plate, scale bar = 500 μm. *n* = 3. (**C**) Each group’s supernatant’s fluorescence intensity was measured. M group represents cells that were treated with M-CSF only. (**D**) Quantification of each group’s relative bone resorption pit area. *** *p* < 0.001 vs. control, ** *p* < 0.01 vs. control.

Prerequisites for osteoclastic bone resorption include modulating fibrous actin cytoskeleton reorganization for the creation of osteoclast sealing zones [28]. The imaging of the intact sealing fibrous actin (F-actin) rings generated in RANKL-induced osteoclasts was made possible by staining actin with rhodamine phalloidin. F-actin ring staining and bone resorption assays were performed to investigate the effect of KCB-F06 on mature osteoclast phenotype and function. As expected, f-actin staining results showed that KCB-F06 decreased the size and number of F-actin rings (Figure 3A). Moreover, bone resorption assay results showed that KCB-F06 treatment groups had significantly fewer resorption pit areas as compared to the control or non-treatment group (Figure 3B,D). Both were performed in a dose-dependent manner. Furthermore, the fluorescence intensity from the bone resorption assay of the treatment groups also showed a similar tendency (Figure 3C). Accordingly, by suppressing the development of mature osteoclasts and the production of F-actin rings, KCB-F06 reduced bone resorption.

### 3.4. The Expression of Osteoclast Differentiation Markers Were INHIBITED by KCB-F06 Treatment

**Figure 4:**
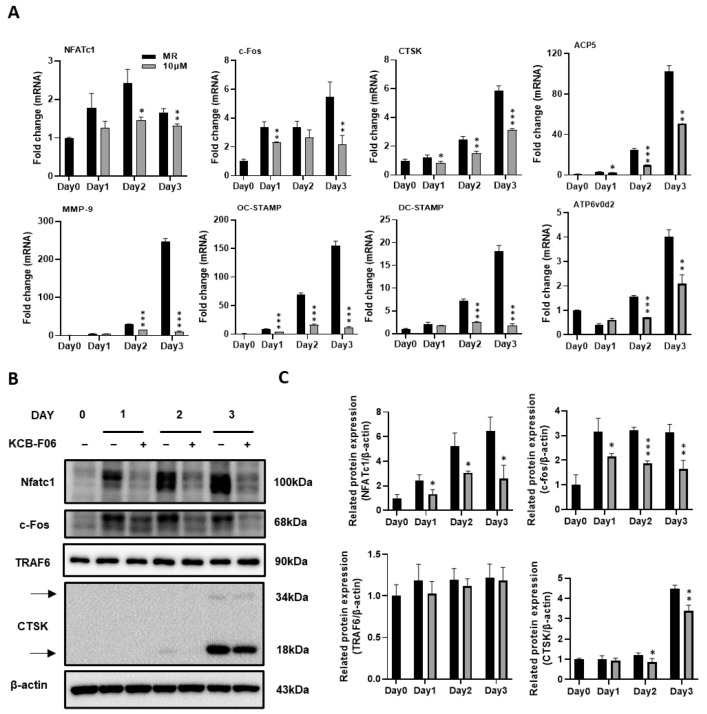
KCB-F06 inhibits osteoclast-specific gene expression during RANKL-induced osteoclastogenesis. (**A**) BMMs were cultured with M-CSF and RANKL with the existence of KCB-F06 for 3 days at the indicated concentration. The mRNA expression levels of NFATc1, c-Fos, cathepsin K (CTSK), MMP9, OC-STAMP, DC-STAMP, ACP5, and ATP6v0d2 were measured by real-time PCR. *n* = 3. (**B**) Western blot assays were performed to detect the protein expression levels of NFATc1, c-Fos, TRAF6, and CTSK. *n* = 3. (**C**) The expression of all the proteins mentioned above was standardized to β-actin expression. *** *p* < 0.001 vs. control, ** *p* < 0.01 vs. control, * *p* < 0.05 vs. control.

During the process (differentiation, fusion, and function) of RANKL-induced osteoclastogenesis, several vital osteoclast-specific markers are upregulated. The effects of KCB-F06 on the regulation of the expression of osteoclast-specific genes and proteins were investigated using real-time PCR and Western blot. Results indicated that KCB-F06 downregulated the mRNA expressions of nuclear factor of activated T-cell c1 (NFATc1), c-Fos, TRAP, DC-STAMP, cathepsin K, OC-STAMP and MMP-9 during RANKL-induced osteoclastogenesis (Figure 4A).

A crucial downstream nuclear transcription factor, NFATc1, regulates the transcription of genes involved in osteoclastic differentiation and function [8,29]. A member of the transcription factor family known as activator protein 1 (AP-1) subunit c-Fos binds cooperatively with NFATc1 in the nucleus [8,29]. Meanwhile, TRAF6 is a direct downstream molecule of RANK [8,29]. By generating protons and lysosomal enzymes, such as Cathepsin K, via a ruffled border, osteoclasts resorb bones by dissolving the bone matrix [28]. Therefore, the protein expression of NFATc1, c-fos, TRAF6, and cathepsin K were examined. As shown in Figure 4B,C, Western blot results demonstrated that KCB-F06 suppressed the protein expressions of NFATc1, c-Fos, and cathepsin K during the process of RANKL-induced osteoclastogenesis. However, TRAF6 protein expression was not influenced by KCB-F06 treatment. These results suggested that KCB-F06 functions downstream of TRAF6 and suppresses osteoclastogenesis by inhibiting the NFATc1 and c-fos mediated signaling.

### 3.5. KCB-F06 Treatment Protects against OVX-Induced Bone Loss

**Figure 5:**
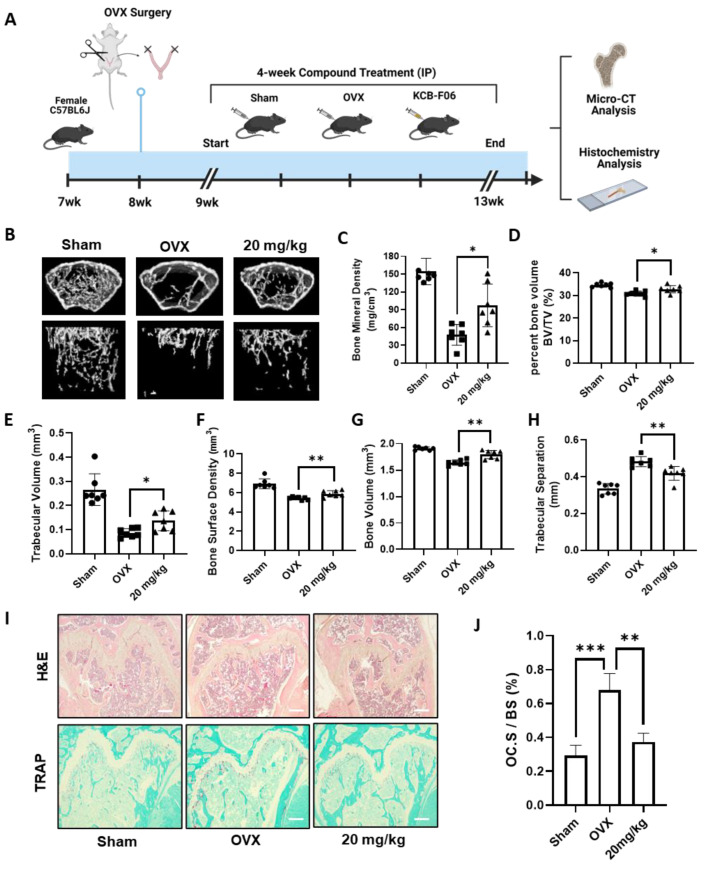
KCB-F06 prevents OVX-induced systematic bone loss in mice. (**A**) An experimental model was designed to investigate the effect of KCB-F06 on the treatment of osteoporosis in OVX mice. (**B**) Micro-CT 3D reconstructed images of the Sham (*n* = 7), OVX (*n* = 7), and KCB-F06 10 mg/kg (*n* = 7) treatment groups in mice. (**C**–**H**) BMD, BV/TV, Tb-V, BSD, BV, and Tb-S of each sample were measured and calculated. (**I**) Representative images of decalcified bone stained with TRAP and H&E are shown, along with a histological assessment of the effects of KCB-F06 (20 mg/kg) on OVX-induced bone loss. (**J**) Quantitative analysis of the percentage of osteoclast surface per bone surface (OC. S/BS) in Panel (**I**). Scale bar = 200 μm. *** *p* < 0.001 vs. control, ** *p* < 0.01 vs. control, * *p* < 0.05 vs. control.

To study the in vivo efficacy of KCB-F06 on managing osteoclastic disorders, an OVX-induced osteoclastic bone loss mouse model was established (Figure 5A). As seen in Figure 5B, micro-computed tomography (micro-CT) analysis of the regions of interest of the femurs demonstrated extensive trabecular bone loss in OVX groups, while it was significantly reversed in the KCB-F06-treated group (Figure 5B), as indicated by the BMD, BV/TV, Tb. V, etc. values (Figure 5C–H). Furthermore, histological analysis using TRAP and H&E staining in Figure 5I showed that KCB-F06 reduced the osteoclast-positive areas and rescued the trabecular bone. The positive areas in each TRAP-stained section were calculated using ImageJ (https://imagej.net/software/fiji/downloads, accessed on 1 December 2023) and compared with the total surface area of the bone. The calculated results are shown in Figure 5J. Thus, KCB-F06 has the potential to be employed as a candidate for developing anti-osteoporosis drugs.

### 3.6. KCB-F06 Activated the Keap1/Nrf2 Signaling Pathway

**Figure 6:**
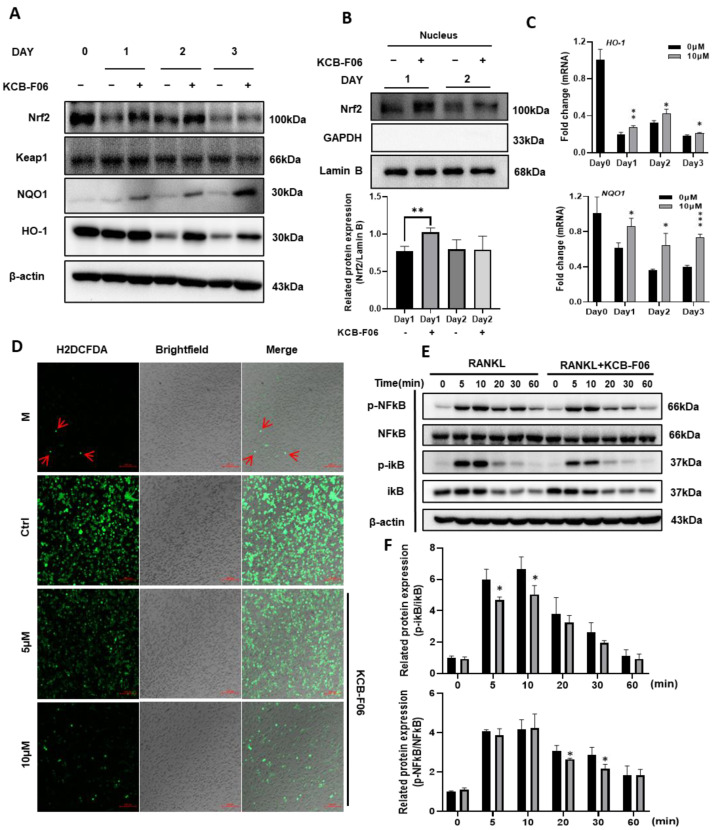
KCB-F06 promotes antioxidant proteins Nrf2 and downstream protein expression and inhibits ROS production during OC differentiation. (**A**) At the specified times of osteoclastogenesis, Expression of Nrf2, Keap1, NQO1, and HO-1 was examined. (days 1, 2, and 3). *n* = 3. (**B**) protein levels of Nrf2 in the nucleus were analyzed by Western blotting. *n* = 3 (**C**) The mRNA expression levels of HO-1 and NQO-1 were measured by real-time PCR. (**D**) BMDMs were seeded on glass slide for 3 days in a differentiation medium in the presence and absence of KCB-F06. ROS generation was detected by DCFDA staining. Scale bar = 100 μm. *n* = 3. (**E**) RANKL was used to activate BMDMs for the specified durations after they were either pretreated with 10 μM KCB-F06 for two hours or not. Cell lysates were examined by Western blotting for p-NFκB, NFκB, p-iκB, iκB, and β-actin. *n* = 3. (**F**) Quantified p-NFκB and p-iκB protein expression levels were normalized to total NFkB and ikB levels. *** *p* < 0.001 vs. control, ** *p* < 0.01 vs. control, * *p* < 0.05 vs. control.

ROS-induced oxidative stress is susceptible to osteoclast formation. The data in Figure 1 suggested that KCB-F06 binds to Keap1, otherwise known as the auto-inhibitor protein of Nrf2. Hence, we explored the Keap1/Nrf2 signaling pathway in osteoclastogenesis. As shown in Figure 6A, KCB-FO6 treatment resulted in an increase in Nrf2 protein expression, whereas Nrf2 expression was greatly reduced in the control group (RANKL-only treatment). Meanwhile, the protein expression of Keap1 was not influenced by the KCB-F06 treatment. The nuclear translocation of Nrf2 was also analyzed, and results suggested that KCB-F06 treatment promoted Nrf2 nuclear translocation (Figure 6B). Since Nrf2 is a key transcription factor for antioxidant proteins involved in ROS accumulation during oxidative stress, the ROS levels with or without KCB-F06 treatment were analyzed. Results showed that KCB-F06 blocked ROS accumulation during RANKL-induced osteoclastogenesis (Figure 6D). Moreover, the protein and mRNA expression levels of the antioxidant proteins HO-1 and NQO1 (Figure 6A,C) increased (The band density quantification is shown in Figure S3). To confirm that the role of Nrf2 activation during KCB-F06-mediated inhibitory activity in osteoclast is essential, a recovery experiment using a known inhibitor of Nrf2 called ML385 was performed (Figure S4). Results showed that ML385 treatment nullifies KCB-F06 activity to enhance the expression of Nrf2 and subsequent inhibition of osteoclastogenesis. Therefore, we can predict that KCB-F06 can block osteoclastogenesis by activating Nrf2-mediated oxidative stress response.

### 3.7. KCB-F06 Inhibited RANKL-Induced NF-κB Signaling Pathway

RANKL stimulation increases intracellular ROS generation during osteoclastogenesis; ROS acts as an upstream element in RANKL signaling cascades to support osteoclast survival and differentiation by triggering NF-κB signaling pathways in osteoclast precursors [10]. The Western blot investigation of the I-κB/NF-κB signaling pathways indicated that KCB-F06 was able to suppress the RANKL-induced phosphorylation of I-κB and p65 NF-kB in pre-osteoclasts (Figure 6E,F).

### 3.8. KCB-F06 Activated Nrf2 by Preventing Its Ubiquitin-Proteasome Degradation via Competitive Binds with Keap1

**Figure 7:**
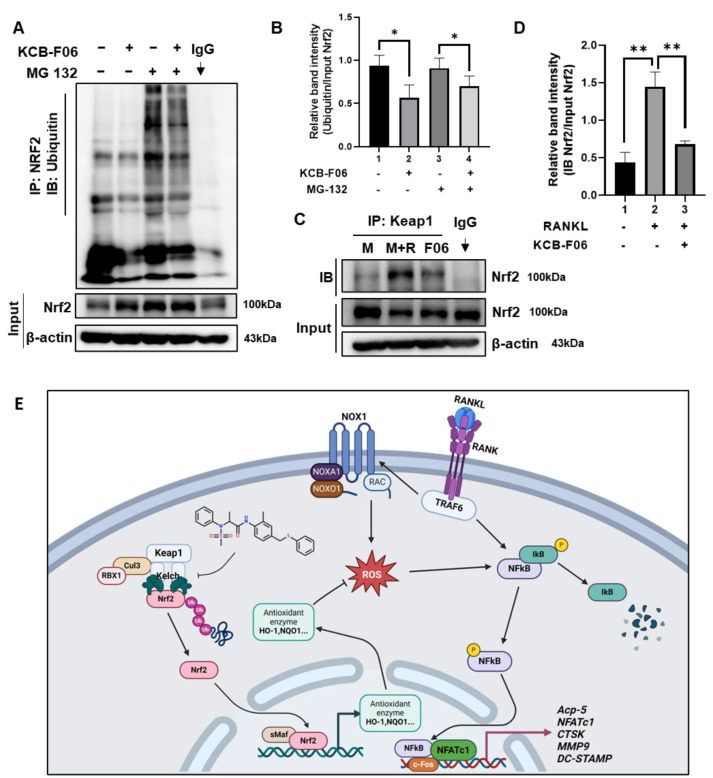
KCB-F06 downregulated the ubiquitination of Nrf2 and influenced the binding between Keap1 and Nrf2 (**A**) BMDMs were treated with M-CSF in the presence and absence of MG 132 and KCB-F06 for 24 h, Nrf2 ubiquitination level was detected by immunoprecipitation assays. *n* = 3. Band intensity was shown in panel (**B**). (**C**) After 24 h of treatment with or without RANKL and KCB-F06, the cell lysate was collected, incubated with Keap1 antibody, and then incubated with protein A/G agarose beads overnight. The supernatant was harvested and Nrf2 levels were analyzed using immunoblotting. *n* = 3. Quantified Nrf2 levels were normalized to input level (**D**). (**E**) This biorender.com created a proposed molecular pathway that explains how KCB-F06 suppresses RANKL-induced osteoclast differentiation. ** *p* < 0.01 vs. control, * *p* < 0.05 vs. control

Under physiological conditions, Nrf2 is degraded in a ubiquitin-proteasome manner by Keap1 [13,19]. Given that KCB-F06 binds to Keap1 (Figure 1) and KCB-F06 blocked osteoclastogenesis through Nrf2 activation, immunoprecipitation assays were performed to determine whether KCB-F06 affected Nrf2 ubiquitination and degradation. As shown in Figure 7A,B, KCB-F06 decreased ubiquitination and degradation of Nrf2, suggesting that KCB-F06 promoted the Nrf2 stability by inhibiting ubiquitin degradation of Nrf2. Due to KCB-F06 binding to Keap1 while not affecting its protein expression (Figure 6A), we came to speculate that KCB-F06 stabilized Nrf2 by disrupting the Keap1-Nrf2 interaction. Immunoprecipitation assays of Keap1 and Nrf2 were performed, and the results suggested that KCB-F06 was able to block the interaction between Keap1 and Nrf2 (Figure 7C,D). These results demonstrated that KCB-F06 is bound tightly to Keap1, and as a result, it can disrupt the Keap1–Nrf2 PPI, consequently preventing Nrf2 ubiquitin-proteasome degradation. Taken together, a proposed molecular mechanism of a KCB-F06 suppressive of RANKL-induced osteoclast differentiation was summarized (Figure 7E).

## 4. Discussion

Bone homeostasis is maintained primarily by a dynamic balance between bone formation and bone resorption [4]. Over-activated mature osteoclasts disrupt the bone microarchitecture, leading to bone loss-linked diseases, such as osteoporosis and rheumatoid arthritis (RA) [7,15]. Current clinically available agents for osteoporosis treatment exhibit side effects after long-term administration, such as long-term treatment of bisphosphonates suppresses bone formation, and results in nonunion after fracture [5]. It has been confirmed that Nrf2 is a promising therapeutic target for a variety of bone disorders. Deficiency of Nrf2, a regulator of cellular redox status, promotes RANKL-induced osteoclastogenesis by controlling the expression of oxidative response genes [13,30]. Additionally, Nrf2 knockout mice suffered a more severe bone destruction in periodontitis and RA models [31]. Recently, several studies suggested that Nrf2 agonists could effectively protect against bone loss by suppressing the activation of osteoclasts [10,13]. Inhibition of Keap1-Nrf2 PPI has been recently suggested as a safe and effective approach to activate Nrf2 for treating ROS and oxidative stress-related diseases [13,14,32]. Hence, we tried to find a novel and effective Keap1-Nrf2 PPI inhibitor and further evaluate its anti-osteoporosis activity to discover a novel anti-osteoclast drug candidate. In this study, AutoDock Vina was used to apply a molecular docking to explore the putative Keap1-Nrf2 PPI inhibitor. (Figure 1A). As a result, KCB-F06 was discovered as a candidate showing great affinity with the binding pocket of Keap1-Nrf2 (Figure 1D,E). Moreover, KCB-F06 inhibited osteoclastogenesis (Figure 1B). Hence, KCB-F06 was selected as a potential effective Keap1-Nrf2 PPI inhibitor. In addition, our DARTS and CETSA assays also confirmed the interaction between KCB-F06 and Keap1 (Figure 1F,G). Next, we demonstrated that KCB-F06 dose and time-dependently inhibited mouse and human osteoclast differentiation in vitro (Figure 2 and Figure S2) as well as suppressed F-actin formation and bone resorption (Figure 3). Therefore, in this study, we screened and discovered a novel Keap1/Nrf2 PPI inhibitor, KCB-F06 that could effectively inhibit osteoclast differentiation in vitro and in vivo. However, one potential limitation of the study is that we were unable to see the role of osteoblasts in the OVX mouse model during in vivo investigations.

Nrf2, a crucial nuclear transcription factor, protects cells from ROS-induced oxidative damage. The regulation of gene expression by Nrf2 translocation into the nucleus affects a group of antioxidant enzymes, such as heme oxygenase-1 (HO-1) and NAD(P)H quinone oxidoreductase 1 (NQO1), which in turn prevent intracellular ROS generation [33]. A careful control of ROS levels is necessary to avoid excessive osteoclastogenesis and the related bone resorption, in addition to ROS’s function in osteoclast differentiation and activation [34]. Our results suggested that KCB-F06 binding to Keap1 leads to the activation of Nrf2. This was confirmed when results shown in Figure 6B suggested that KCB-F06 enabled the promotion of Nrf2 nuclear translocation. In addition, the expression levels of Keap1 were not influenced, while the protein levels of Nrf2 increased after KCB-F06 treatment. Meanwhile, the protein and mRNA expressions of HO-1 and NQO1 in were significantly upregulated after KCB-F06 treatment (Figure 6A,C and Figure S4). The ROS accumulation during osteoclast differentiation was also dose-dependently inhibited by KCB-F06 (Figure 6D).

The binding of RANKL to RANK is known to induce the recruitment of TRAF6, which stimulates the downstream I-κB/NF-κB signaling pathway to regulate c-fos and NFATc1, both required for osteoclastogenesis [35]. In this study, KCB-F06 treatment showed no significant influence on the expression of TRAF6, but it remarkably attenuated RANKL-mediated c-fos and NFATc1 expression (Figure 4B,C). Given that the I-κB/NF-κB signal pathway is a famous signaling sensitive to intracellular ROS levels and KCB-F06 inhibited ROS levels during osteoclastogenesis (Figure 6D), we inferred that KCB-F06 suppressed the ROS-mediated I-κB/NF-κB signal pathway to regulate osteoclastogenesis (Figure 6E,F). Furthermore, due to the increased Nrf2 protein levels and the activated antioxidant target genes (HO-1 and NQO1) downstream of Nrf2 after KCB-F06 treatment during osteoclastogenesis, we can conclude that KCB-F06 indeed enhanced Nrf2 activation during osteoclast differentiation. Hence, KCB-F06 inhibited at least partly osteoclast differentiation through Nrf2 and its downstream pathways.

Intracellular levels of Nrf2 are regulated by ubiquitination and subsequent proteasome-mediated protein degradation [9,19]. Our results in Figure 1 suggested that KCB-F06 binds to Keap1 without interfering with its protein levels (Figure 6A). Therefore, we hypothesized that the mechanism by which KCB-F06 regulates Nrf2 protein levels is by preventing the ubiquitin degradation of Nrf2 by disturbing the Keap1-Nrf2 PPI. To confirm this hypothesis, we examined the effect of KCB-F06 on ubiquitin-Nrf2 protein levels with or without treating MG 132, a known proteasome inhibitor (Figure 7A,B). The interaction between Keap1 and Nrf2 after KCB-F06 treatment was also checked through immunoprecipitation assays (Figure 7C,D). The results illustrated that KCB-F06 indeed stabilizes Nrf2 protein by disturbing Keap1/Nrf2 interaction to suppress ubiquitin-proteasome-mediated Nrf2 protein degradation.

According to recent reports, several ROS inhibitors, such as 4-methylatechol, Oroxylin A, and Notopterol, can inhibit osteoclast activity while exerting anti-osteoporosis effects through antioxidant pathways similar to KCB-F06 [36,37,38]. In particular, 4-methylatechol works through the ROS/Keap1/Nrf2 signaling axis, and Keap1 is the predicted target, indicating that regulating ROS is a feasible and effective approach. Moreover, compared to these natural chemicals, KCB-F06 has the best IC_50_ culturing with osteoclasts in terms of dose range. From this point of view, KCB-F06 can be considered as a great anti-osteoporosis candidate.

In summary and consistent with previous reports that suggested that Nrf2 activation could alleviate osteoclastic bone loss [10], our study revealed that KCB-F06, a novel Keap1-Nrf2 PPI inhibitor, repressed mature osteoclast formation, decreased bone resorption function in vitro, and attenuated OVX-induced bone loss in vivo. In addition, KCB-F06 disturbed Keap1/Nrf2 interaction to activate the Nrf2-mediated antioxidative response gene. This led to a reduction in ROS accumulation, which therefore, repressed I-κB/NF-κB signaling activation, and downregulated NFATc1 and c-fos expressions. Thus, we were able to demonstrate KCB-F06 as a prospective therapeutic candidate against osteoporosis.

## Data Availability

Data are contained within the article.

## Supplementary Materials

The following supporting information can be downloaded at https://www.mdpi.com/article/10.3390/antiox13070850/s1.
