# Supplementary material for: Novel Inhibitor of Keap1-Nrf2 Protein–Protein Interaction Attenuates Osteoclastogenesis In Vitro and Prevents OVX-Induced Bone Loss In Vivo
Source: Antioxidants (Basel). 2024 Jul 15;13(7):850. doi: 10.3390/antiox13070850 (PMC11273523; doi:10.3390/antiox13070850)
Supplement: Supplementary file 1 [file antioxidants-13-00850-s001.zip › antioxidants-3097337-supplementary.pdf]

Table of Contents

1. Table S1 List of primers.....S-2
2. Figure S1 KCB-F06 mainly inhibited during the middle stage of osteoclast differentiation.....S-3
3. Figure S2 KCB-F06 showed no effect on osteoblast differentiation.....S-4
4. Figure S3 Antioxidant-related protein band density quantification.....S-5
5. Figure S4 ML385 promoted osteoclastogenesis and eliminated the effect of KCB-F06 on osteoclasts.....S-6

Table S1. List of primers.

Gene	Gene Accession Numbe	Primer sequence (5′→3′)	
Nfatc1	NM_001164112	Forward	ACCACCTTTCCGCAACCA
		Reverse	GGTACTGGCTTCTCTTCCGTTTC
c-fos	NM_010234	Forward	CGAAGGGAACGGAATAAGATG
		Reverse	GCTGCCAAAATAAACTCCAG
Cathepsin k	NM_007802	Forward	ACTTCCGCAATCCTTACCGA
		Reverse	TTCGCTAGGCTCTTTTCGGA
Acp5	NM_001102405	Forward	TTTATGCTGGACACAGTGATGCT
		Reverse	CCCAGGTCTCGAGGCATTT
Mmp9	NM_174744	Forward	CTGGACAGCCAGACACTAAAG
		Reverse	CTCGCGGCAAGTCTTCAGAG
Oc-stamp	NM_02902	Forward	ACTATGGCCACCCGGGAAT
		Reverse	GGCCCAAGGGAGTCATGTG
Dc-stamp	NM_029422.4	Forward	CGCACGATGCTTCATTCTTC
		Reverse	CAGTGCCAGCCGCAATC
Atp6v0d2	NM_175406	Forward	GTGAGACCTTGGAAGACCTGAAA
		Reverse	TCCTCATCTCCGTGTCAATTTTG
Gapdh	NM_00128972	Forward	TGTGTCCGTCGTGGATCTGA
		Reverse	GATGCCTGCTTCACCACCTT
HO-1	NM_010442	Forward	CCCACCAAGTTCAAACAGCTC
		Reverse	AGGAAGGCGGTCTTAGCCTC
NQO1	NM_008706	Forward	TTCTGTGGCTTCCAGGTCTT
		Reverse	TCCAGACGTTTCTTCCATCC

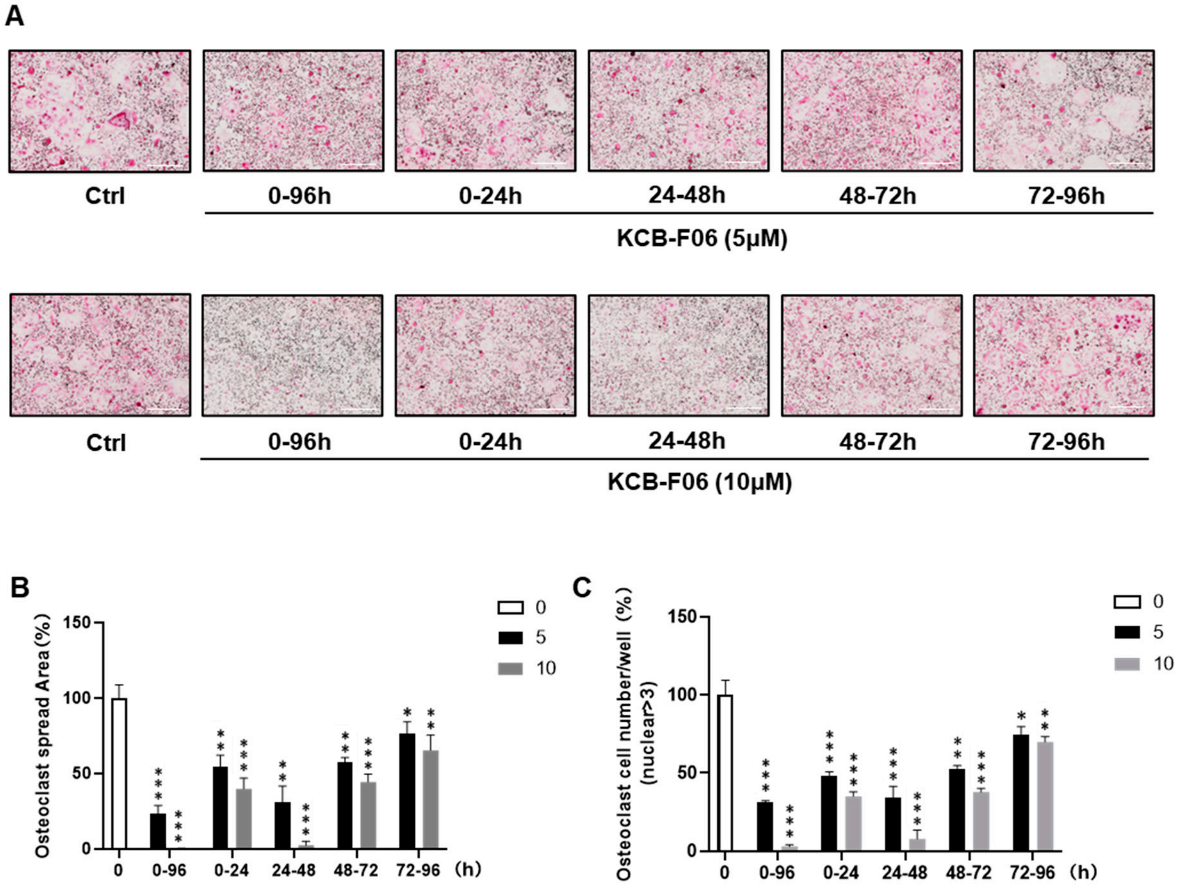

Figure S1. KCB-F06 mainly inhibited during the middle stage of osteoclast differentiation. (**A**) BMMs were divided into five groups, each group grown in culture medium containing M-CSF and RANKL for 4 days and were exposed to KCB-F06 for 24 h on different days, respectively. Representative images of TRAP staining were shown. Scale bar = 1mm. (**B**) The quantitative analysis of TRAP-positive multinucleated cell area (nuclei >3) of the five groups and (**C**) their osteoclast cell number. *n*=4. *** $p<0.001$ vs. control, ** $p<0.01$ vs. control, * $p<0.05$ vs. control.

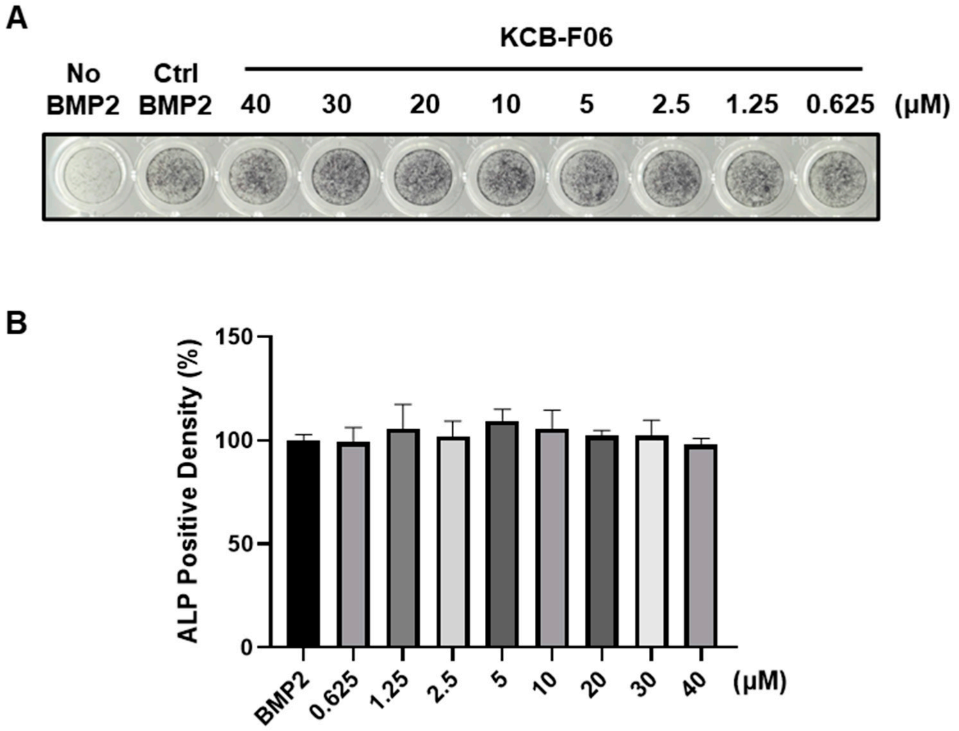

Figure S2. KCB-F06 showed no effect on osteoblast differentiation. (**A**) ALP staining was performed on day 9 following osteoblast differentiation induced by the addition of 100 ng/mL BMP2, along with the designated concentrations of KCB-F06. (**B**) The intensity of ALP staining of each group was quantified using ImageJ. *n*=4.

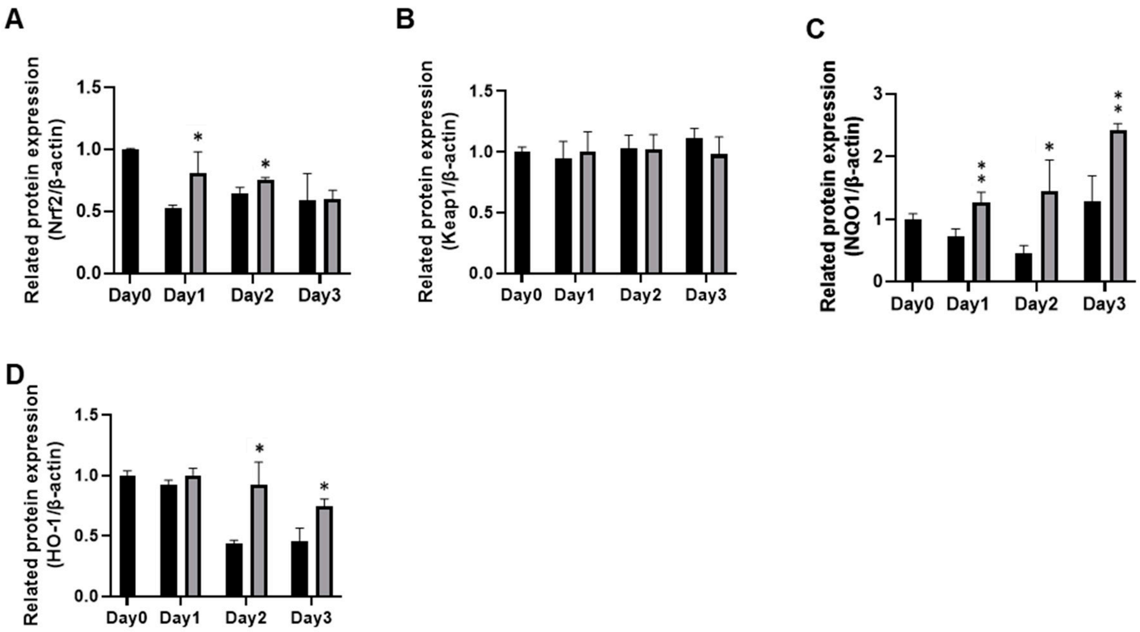

Figure S3. Antioxidant-related proteins band density quantification. Protein quantification graph of Figure 6A. (**A**) to (**D**) Band intensities of the indicated proteins in Figure 6A were calculated using ImageJ. *n*=3. ** $p<0.01$ vs. control, * $p<0.05$ vs. control.

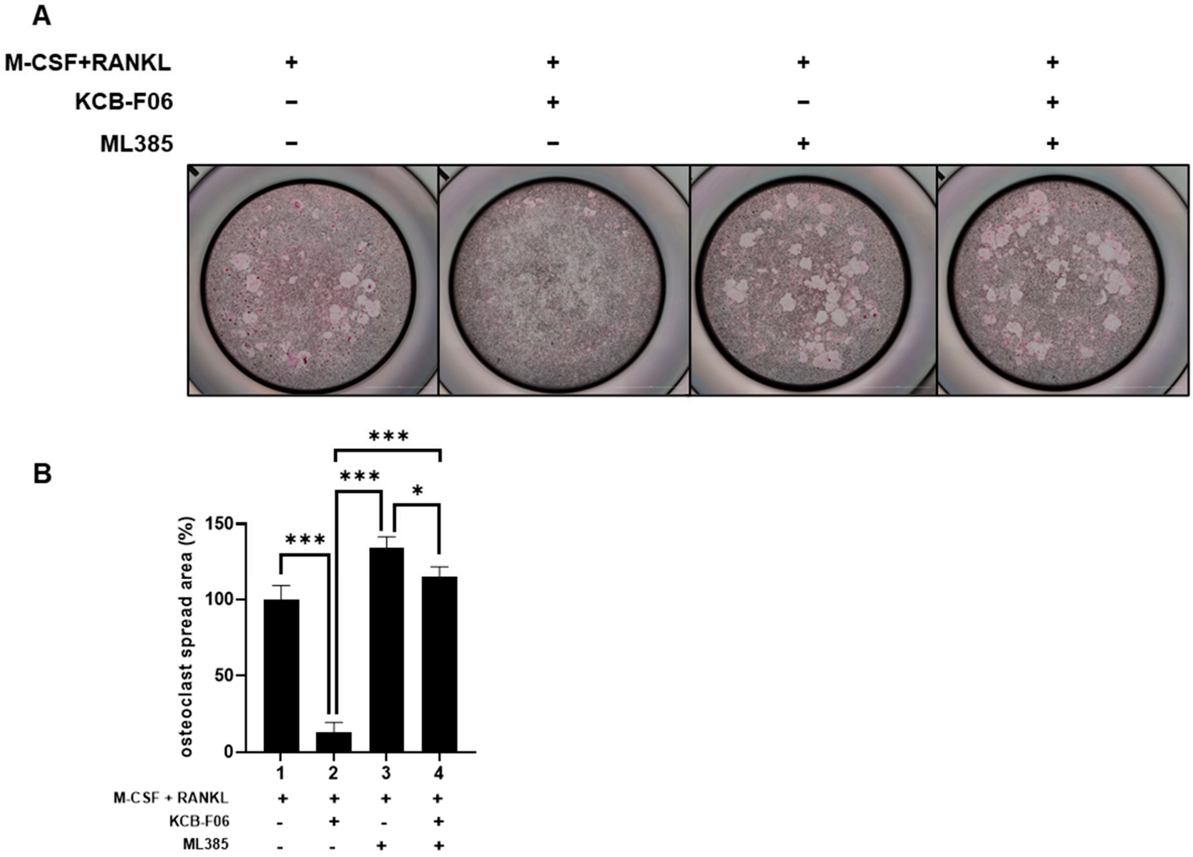

Figure S4. ML385 promoted osteoclastogenesis and eliminated the effect of KCB-F06 on osteoclasts. (**A**) BMMs were cultured with or without M-CSF, RANKL, ML385 (0.625 μM) and KCB-F06 (5 μM). The cells were incubated in 96-well plates and after 3 days, TRAP staining was performed. (**B**)The quantitative analysis of TRAP-positive multinucleated cells area (nuclei >3) of the four groups. *n*=4. *** p<0.001 vs. control, * p<0.05 vs. control.
